# Research on the Sensitivity Enhancement Method of Inductive Conductivity Sensors Based on Impedance Matching

**DOI:** 10.3390/s25020293

**Published:** 2025-01-07

**Authors:** Yang Li, Rongxing Yu, Zhiqiang Liu, Yujie Gao, Chengxu Tang, Qiao Li, Chao Yuan

**Affiliations:** 1 EHV Transmission Companies Dali Office of China Southern Power Grid Co., Ltd., Dali 671000, China; liyang2@im.ehv.csg (Y.L.); yurongxing1@im.ehv.csg (R.Y.); liuzhiqiang@im.ehv.csg (Z.L.); gaoyujie@im.ehv.csg (Y.G.); 2 College of Electrical and Information Engineering, Hunan University, Changsha 410082, China; tcx22@hnu.edu.cn (C.T.); qiaoli@hnu.edu.cn (Q.L.)

**Keywords:** inductive conductivity sensor, impedance matching, sensitivity enhancement, signal optimization

## Abstract

This paper presents the design and performance evaluation of an inductive conductivity sensor with a double tuning impedance matching network to enhance sensitivity and improve linearity. The sensor’s equivalent circuit model is analyzed and verified through simulation, and impedance matching is shown to significantly increase the sensor’s output signal, particularly at low conductivity measurements. Double tuning impedance matching expands the frequency response range and optimizes power transfer efficiency, achieving a higher power factor across a broader frequency range. Experimental results confirm that the sensor’s sensitivity increases by approximately 30% after impedance matching with optimal performance at a frequency of 9865 Hz. Furthermore, while the impedance matching improves sensitivity, it also introduces some nonlinear errors, which are evaluated using a performance function that balances sensitivity and linearity. The results demonstrate that impedance matching enhances the sensor’s measurement capability, making it more effective in practical applications where conductivity changes need to be accurately monitored across varying frequencies.

## 1. Introduction

Conductivity sensors are widely used in environmental monitoring, chemical process control, and water treatment with their primary function being the measurement of solution conductivity to assess water quality [1,2,3,4]. In power systems, particularly in high-voltage direct current (HVDC) converter valve cooling systems, conductivity sensors play a critical role by continuously monitoring the conductivity of cooling water. Excessive conductivity can lead to sedimentation and scaling, which may compromise system efficiency and the reliability of electrical equipment [5,6,7].

According to their measurement principles, conductivity sensors can be broadly classified into three types: electrode type, inductive type, and capacitive type. Electrode-type conductivity sensors apply a voltage between electrodes and measure the current in the fluid to calculate conductivity based on Ohm’s law [8]. However, due to the direct contact between the electrodes and the liquid, this type of sensor is susceptible to issues such as contamination, polarization, and electrode aging, which limits its measurement stability and accuracy. Inductive conductivity sensors, on the other hand, use electromagnetic induction to achieve non-contact measurement, effectively avoiding the inherent defects of electrode-type sensors [9]. This non-contact measurement method not only significantly enhances the long-term stability of the sensor but also makes it particularly suitable for environments with high contamination or applications that require extremely high measurement precision, such as complex industrial water treatment systems and precise measurement tasks [10,11,12]. Furthermore, the structural design of inductive sensors can accommodate corrosive liquids and complex operating conditions, further improving their reliability and applicability. Capacitive conductivity sensors use changes in the electric field for non-contact measurement, which also avoids contamination and polarization issues [13]. They perform better in certain specific scenarios, such as measuring highly corrosive liquids. However, their performance may be limited by variations in the dielectric constant of the liquid, negatively impacting measurement accuracy and stability. In contrast, the measurement principle of inductive conductivity sensors is not affected by changes in dielectric constant, offering superior environmental adaptability and long-term reliability. With their excellent overall performance and broad applicability, inductive conductivity sensors have become the preferred choice in complex industrial environments and high-precision applications. However, a significant limitation of inductive conductivity sensors is their relatively low output signal, which restricts their performance in high-precision and rapid-response applications. Low output signals reduce measurement sensitivity and make the sensor more susceptible to noise, leading to decreased accuracy. Therefore, enhancing the output signal sensitivity of inductive conductivity sensors has become a critical challenge.

Several researchers have explored methods to improve the output signal of inductive sensors. Wu et al. [14] derived an analytical expression for sensor output based on its principles, identifying key factors influencing performance and proposing methods to enhance the signal, which were validated experimentally. Ashokan et al. [15] investigated the effects of different coil turn ratios and core materials on inductive conductivity sensors. Their experiments identified the optimal operating frequency and voltage response, achieving good temperature dependence and anti-interference capabilities. However, their work did not address how to enhance long-term stability in extreme environmental conditions, which remains an ongoing challenge. Jang et al. [16] analyzed the effects of geometric parameters on the cell constant of inductive conductivity sensors using the finite element method. While their research provided valuable theoretical insights and experimental verification, further studies are needed, particularly under varying operational conditions, such as in complex marine environments. Kandur et al. [17] explored low-cost inductive conductivity sensors, focusing on measurement uncertainty, including hysteresis errors and temperature effects. While these sensors demonstrated good linearity and reproducibility, they still struggled with accuracy under extreme conditions, such as high salinity or fluctuating temperatures. These studies underscore the ongoing efforts to improve inductive conductivity sensor performance, yet significant challenges remain, especially in achieving high precision and stability in demanding environments.

Building on this background, the paper proposes the application of impedance matching technology to optimize the output performance of inductive conductivity sensors. Impedance matching, widely used in signal transmission systems, maximizes power transfer and minimizes losses by adjusting the impedance between the load and the source. In the context of inductive conductivity sensors, proper impedance matching between the sensor and the external circuit can significantly enhance the sensor’s output signal, improving measurement sensitivity and resistance to interference. This approach not only enhances the performance of inductive conductivity sensors but also broadens their potential for high-precision monitoring applications. The objective of this paper is to systematically evaluate the role of impedance matching in enhancing the output signal of inductive conductivity sensors through both experimental and theoretical analysis, ultimately providing an optimized design solution.

## 2. Measurement Principle and Equivalent Circuit of Inductive Conductivity Sensor

### 2.1. Measurement Principle of the Inductive Conductivity Sensor

The measurement principle of inductive conductivity sensors is based on Faraday’s law of electromagnetic induction, enabling the non-contact detection of liquid conductivity. The sensor probe typically consists of two primary components: an excitation coil and a sensing coil, together forming an electrode-free measurement structure. This configuration effectively eliminates the interference from electrode contamination and polarization effects, ensuring long-term stability and reliable performance. When alternating current is applied to the excitation coil, it generates a time-varying magnetic field. This magnetic field induces eddy currents in the conductive medium, which in turn generate a secondary magnetic field that is detected by the sensing coil. The magnitude of the induced current is directly proportional to the conductivity of the liquid, allowing the conductivity to be measured without direct contact with the fluid.

**Figure 1:**
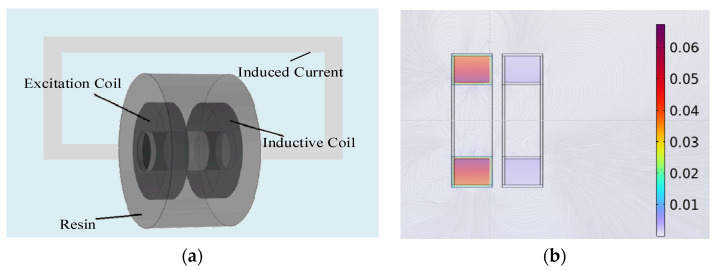
Inductive conductivity sensor. (**a**) Probe structure of inductive conductivity sensor; (**b**) magnetic field distribution during sensor operation.

The structure of the inductive conductivity sensor probe is shown in Figure 1a. According to Faraday’s law of electromagnetic induction, the application of alternating current to the excitation coil produces an alternating magnetic field that extends through the sensor and into the surrounding liquid. This magnetic field induces eddy currents (or induced currents) in the conductive medium, the strength of which is directly related to the liquid’s conductivity. The induced currents generate a secondary alternating magnetic field within the liquid, which induces an electromotive force (EMF) in the sensing coil. Since the conductivity of the liquid determines the strength of the induced current, it directly influences the output signal of the sensing coil. By measuring the amplitude and frequency characteristics of the signal from the sensing coil, the conductivity of the liquid can be inferred. Figure 1b shows the magnetic flux density distribution of the sensor probe, which is obtained through finite element simulation. The excitation coil magnetically couples with the sensing coil through the conductive liquid, inducing magnetic flux in the magnetic core of the sensing coil. This, in turn, generates an electromotive force (EMF) in the sensing coil, which is then used to measure the conductivity.

**Figure 2:**
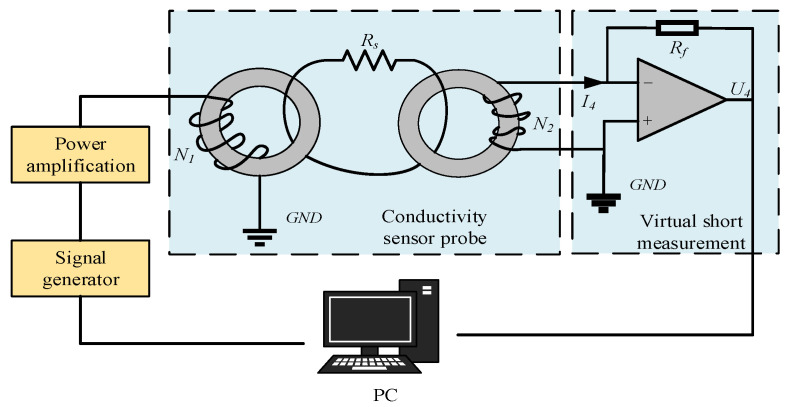
Sensor measurement principle diagram.

A virtual short-circuit circuit, constructed using an operational amplifier, is employed to measure the sensor output, as shown in Figure 2. The excitation signal, generated by the signal generator and power amplification module, is applied to the excitation coil. The measured liquid is modeled as a single-turn coil, with its conductivity represented by a resistance *R_s_*, which is placed between the excitation coil and the sensing coil, creating a coupling mechanism that generates an output signal at the sensing coil. Due to the virtual short-circuit property of the operational amplifier, the output of the sensing coil is effectively short-circuited. The short-circuit current *I*_4_ is influenced by the conductivity of the measured liquid, the geometry of the sensor, and the excitation frequency. Once the sensor’s structure and operating conditions are fixed, the short-circuit current *I*_4_ becomes directly proportional to the conductivity of the measured liquid. Exploiting the virtual open-circuit property of the operational amplifier, the short-circuit current *I*_4_ flows through the feedback resistor *R_f_*, following the relationship *U_out_* = *R_f_* × *I*_4_. Therefore, by measuring the output voltage *U_out_* of the operational amplifier, the conductivity of the measured liquid can be determined.

### 2.2. Equivalent-Circuit Model of the Inductive Conductivity Sensor

**Figure 3:**
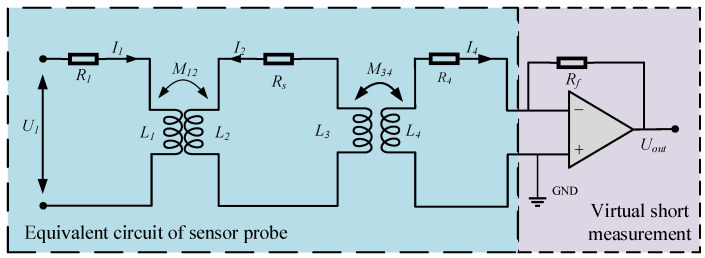
Equivalent circuit model of inductive conductivity sensor.

The equivalent circuit of the inductive conductivity sensor is shown in Figure 3 [18]. The sensor consists of an excitation coil and a sensing coil with electromagnetic coupling established through the measured liquid. In the equivalent circuit, the coupling between the excitation coil, the liquid, and the sensing coil is modeled as the coupling of two ideal transformers. The excitation coil and the sensing coil are magnetically coupled through the conductivity of the liquid. In Figure 3, *L*_1_ and *L*_4_ represent the inductance of the excitation coil and the sensing coil, respectively, while *L*_2_ and *L*_3_ represent the equivalent inductance of the liquid. The mutual inductance between the coils and the liquid is represented by *M*_12_ and *M*_34_. However, due to shielding measures typically implemented between the excitation coil and the sensing coil, the mutual inductance between the two coils is generally neglected in this model. The resistances *R*_1_ and *R*_2_ correspond to the loss resistances of the excitation coil and the sensing coil, respectively. The conductivity of the measured liquid is modeled as a resistance *R_s_* in the circuit. *U*_1_ represents the input voltage applied to the sensor [19].

To obtain the output electromotive force (EMF) of the sensor, the following equation can be derived by applying Kirchhoff’s law:

(1) $$\left\{\begin{array}{c} j\omega M_{12}I_{2}+I_{1}\left(R_{1}+j\omega L_{1}\right)=U_{1}\\ j\omega M_{12}I_{1}+j\omega M_{34}I_{4}+I_{2}\left(R_{s}+j\omega L_{2}+j\omega L_{3}\right)=0\\ j\omega M_{34}I_{2}+I_{4}\left(R_{4}+j\omega L_{4}\right)=0 \end{array}\right.$$

Since the magnetic permeability of the sensor core is significantly higher than that of the liquid, it is reasonable to assume perfect coupling between the transformer components in the equivalent circuit model [20,21]. Given that the sensor operates under high-frequency conditions, it can be assumed that *R*_1_ << *ωL*_1_, *R*_4_ << *ωL*_4_, where *ω* is the angular frequency and *L*_1_, *L*_4_ are the inductances of the excitation and sensing coils, respectively. Furthermore, in this study, a virtual short-circuit configuration, implemented with an operational amplifier, is used to measure the short-circuit current. Based on these assumptions, the following conditions can be derived:

(2) $$\left\{\begin{array}{c} R_{1}\ll \omega L_{1},R_{4}\ll \omega L_{4}\\ M_{12}=k_{12}\sqrt{L_{1}L_{2}},M_{34}=k_{34}\sqrt{L_{3}L_{4}}\\ k_{12}=k_{34}=1 \end{array}\right.$$

In the equation, *k*_12_ and *k*_34_ represent the inductive coupling coefficients between the respective coils and the liquid. Therefore, the short-circuit current *I*_4_ can be expressed as

(3) $$I_{4}=\frac{U_{1}\sqrt{{L_{1}L_{2}L_{3}L}_{4}}}{R_{s}L_{1}L_{4}}$$

**Figure 4:**
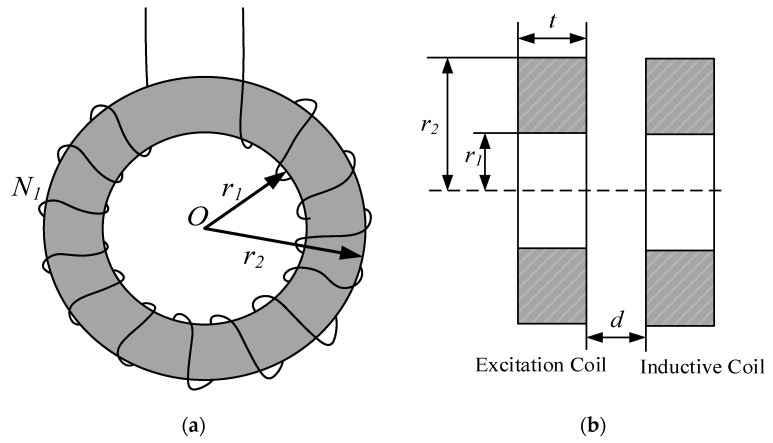
Coil with toroidal magnetic core. (**a**) Structure; (**b**) magnetic ring cross-section.

The probe section of the inductive conductivity sensor comprises two toroidal cores, which are each independently wound with a coil. A schematic diagram of this structure is shown in Figure 4.

In Figure 4, *N*_1_ represents the number of turns of the excitation coil, while *r*_1_ and *r*_2_ are the inner and outer radii of the toroidal magnetic core, respectively. *t* denotes the thickness of the magnetic core, and *d* is the distance between the excitation coil and the sensing coil. Both the excitation coil and the sensing coil have identical physical structures, meaning they share the same number of turns and the same magnetic core dimensions. The inductance *L* of the toroidal coil is given by the formula in Equation (4).

(4) $$L=\frac{\mu_{0}\mu_{r}t}{2\pi }ln\left(\frac{r_{2}}{r_{1}}\right)N^{2}=aN^{2}$$

In the equation, *μ*_0_ represents the permeability of free space, *μ_r_* is the relative permeability of the magnetic core, and *a* is an inductance coefficient that depends on the dimensions and material properties of the toroidal magnetic core.

Substituting Equation (4) into (3) yields

(5) $$I_{4}=\frac{\sqrt{a_{1}a_{2}a_{3}a_{4}}U_{1}}{a_{1}a_{4}N_{1}N_{4}R_{s}}=\frac{\sqrt{a_{1}a_{2}a_{3}a_{4}}U_{1}}{a_{1}a_{4}N_{1}N_{4}k}G_{s}$$

In the equation, *k* is the conductivity constant, *G_s_* represents the conductivity of the water, *a*_1_ is the inductance coefficient of the excitation coil on the toroidal magnetic core, and *a*_4_ is the inductance coefficient of the sensing coil on the toroidal magnetic core. *a*_2_ is the inductance of the water-equivalent single-turn coil on the excitation coil’s toroidal magnetic core, and *a*_3_ is the inductance of the water-equivalent single-turn coil on the sensing coil’s toroidal magnetic core. Since the physical structures of the excitation and sensing coils are identical, it follows that *a*_2_ = *a*_3_. After the short-circuit current *I*_4_ passes through the virtual short-circuit circuit constructed with the operational amplifier, the output voltage *U_out_* is given by *U_out_* = *R_f_* × *I*_4_. Therefore, the sensor’s output can be expressed as

(6) $$U_{out}=\frac{\sqrt{a_{1}a_{2}a_{3}a_{4}}U_{1}R_{f}}{a_{1}a_{4}N_{1}N_{4}k}G_{s}$$

It can be observed that once the input signal, magnetic core coil physical structure parameters, and material properties are determined, the output of the inductive conductivity sensor shows a linear relationship with the conductivity of the measured water.

## 3. Sensitivity Enhancement of Conductivity Sensors Through Impedance Matching

Impedance matching refers to the condition where the load impedance matches the internal impedance of the signal source, thereby maximizing the power transmitted to the load. In a purely resistive circuit, impedance matching occurs when the source impedance equals the load impedance; otherwise, mismatching leads to inefficiencies. For non-resistive circuits, the matching condition requires the load impedance to be the complex conjugate of the source impedance, ensuring optimal power transfer and minimal losses [22,23,24]. In the context of inductive conductivity sensors, impedance matching improves signal transmission efficiency, reducing losses along the signal path. This, in turn, enhances the signal-to-noise ratio (SNR) of the sensor, minimizing the impact of noise in complex environments. As a result, impedance matching allows the sensor to maintain higher sensitivity and faster response even under high-frequency operating conditions [25,26].

### 3.1. Static Impedance Matching

**Figure 5:**
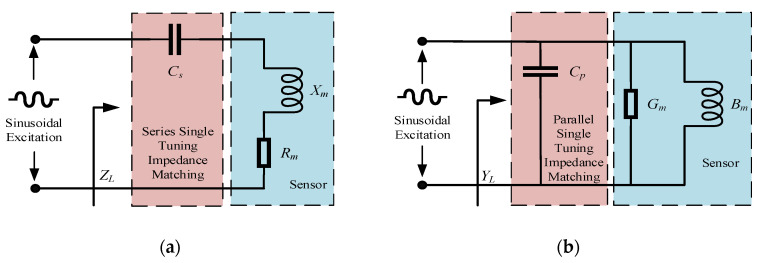
Single tuning static impedance matching circuit. (**a**) Series single tuning impedance matching; (**b**) parallel single tuning impedance matching.

Based on the equivalent circuit model, the inductive conductivity sensor can be represented as a reactive load over the entire frequency range. In practice, the impedance (or admittance) function is measured at the frequency of interest to determine the combined values of resistance (or conductance) and reactance (or susceptance). This leads to a simplified equivalent circuit at the specific frequency, as shown in Figure 5. The series and parallel equivalent circuits can be interconverted. Since the inductive conductivity sensor typically exhibits inductive behavior across the entire frequency range in a static state, the reactance *X* is positive, and the admittance *B* is negative. By adjusting the load impedance with series or parallel capacitors, resonance can be achieved. At the resonant frequency, a unit power factor output from the power supply can be attained, thereby improving the sensor’s performance.

Since the matching capacitor is directly connected to the single-phase AC operating circuit, it must be a high-voltage, non-polarized capacitor. The capacitance value and voltage rating of the capacitor determine its size. In series capacitor matching, the required capacitance is larger compared to parallel matching, but the voltage rating requirement for the capacitor is lower. Additionally, for the same current flowing through the sensor, the external input voltage in series capacitor matching is significantly lower than that in parallel matching. This reduces the demand on the external power supply output voltage. Therefore, series capacitor matching is generally preferred.

Based on the equivalent circuit model of the inductive conductivity sensor, the following expression can be derived:

(7) $$\left\{\begin{array}{c} R_{m}=\frac{\omega^{2}L_{1}L_{2}R_{s}}{\omega^{2}L_{2}^{2}+R_{s}^{2}}\\ L_{m}=\frac{L_{1}R_{s}^{2}}{\omega^{2}L_{2}^{2}+R_{s}^{2}} \end{array}\right.$$

When using series single tuning matching, the equivalent impedance of the circuit is expressed as

(8) $$Z_{L}=R_{m}+j\left(\omega L_{m}-\frac{1}{\omega C_{s}}\right)$$

At the sensor’s operating frequency *ω_m_*, the required matching capacitance *C_s_* is

(9) $$C_{s}=\frac{1}{\omega_{m}^{2}L_{m}}$$

**Figure 6:**
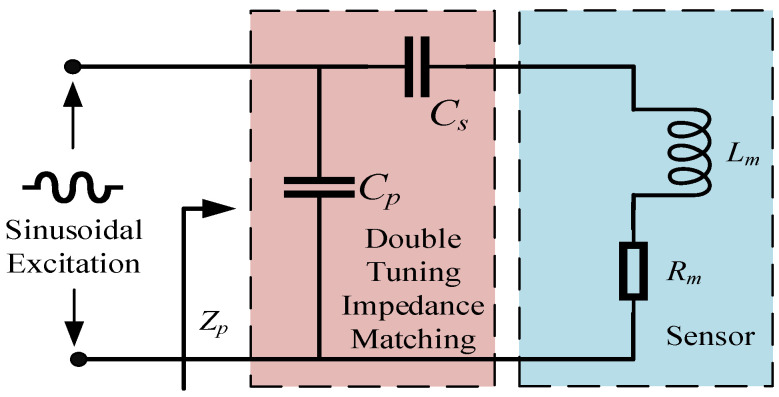
Double tuning static impedance matching circuit.

Single tuning static impedance matching is a commonly used technique to maximize the power transfer between the sensor and the external circuit by achieving a high power factor at a specific frequency. However, this approach is limited by its narrow bandwidth, meaning it is effective only around the resonant frequency. This narrow bandwidth can restrict the sensor’s performance across a wide range of frequencies, especially in applications where the input signal frequency varies. To overcome this limitation, double tuning static impedance matching has been proposed. This method involves designing two resonant peaks at distinct frequency points rather than relying on a single resonant frequency. By adjusting the positions of these two resonant frequencies, a broader frequency passband can be achieved, allowing the system to match impedance more effectively over a wider range of frequencies. The broader passband ensures that the sensor operates efficiently not only at one specific frequency but also across a range of frequencies, thereby improving its performance and adaptability to varying operating conditions. Double tuning static impedance matching enables the sensor to maintain high efficiency and an improved signal-to-noise ratio across a broader frequency spectrum, which is particularly beneficial for applications where the frequency of the input signal can fluctuate. This method allows for more flexible system design, especially in environments with varying operating conditions or when the sensor needs to respond quickly to changes in signal frequency. As illustrated in Figure 6, double tuning impedance matching offers significant advantages in terms of bandwidth expansion while maintaining the power transfer efficiency.

When using double tuning static impedance matching, the passband is defined by the frequencies *ω*_1_ and *ω*_2_ (*ω*_1_ < *ω*_2_), which represent the lower and upper frequencies at which resonance occurs. This frequency range defines the bandwidth within which the sensor operates efficiently, ensuring optimal power transfer. The passband is crucial for determining the overall performance of the sensor in varying signal environments. By selecting appropriate values for *ω*_1_ and *ω*_2_, the system can be tailored to meet specific application requirements, whether for narrow or wideband applications. In this case, the parallel matching capacitance *C_p_* can be expressed as

(10) $$C_{s}=\frac{1}{\omega_{m}^{2}L_{m}}$$

### 3.2. Simulation Analysis

In this work, the equivalent circuit model of the inductive conductivity sensor was simulated and verified using Multisim14 circuit simulation software. The circuit simulation model, based on the equivalent circuit shown in Figure 3, was developed in Multisim to analyze the effect of varying equivalent resistance values *R_s_* on the sensor output. The simulation aimed to understand how changes in the conductivity of the measured liquid (represented by *R_s_*) influence the sensor’s performance, particularly its output signal. The structural parameters of the sensor probe were set according to the specifications listed in Table 1, ensuring consistency with the experimental setup. This simulation served as a critical tool in evaluating the sensor’s behavior under different operating conditions and provided valuable insights into optimizing sensor design for improved performance.

**Figure 7:**
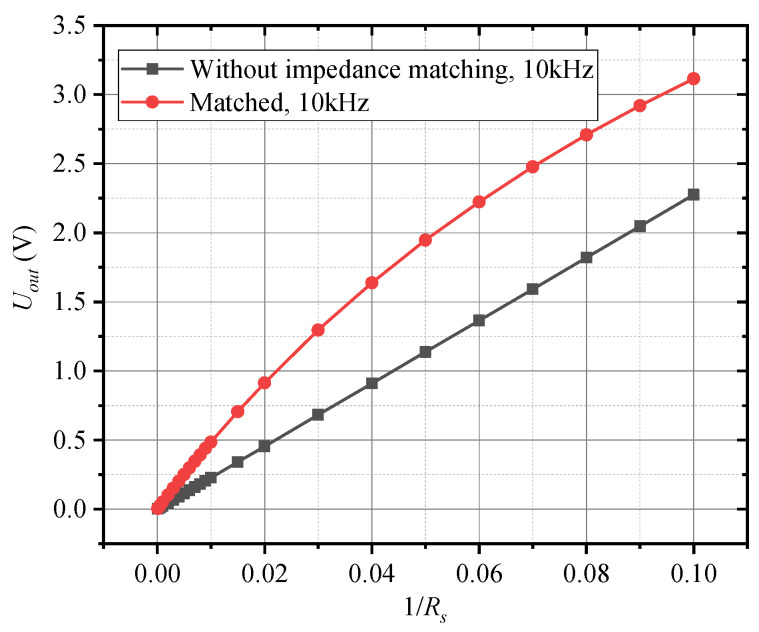
Output response of sensors.

**Figure 8:**
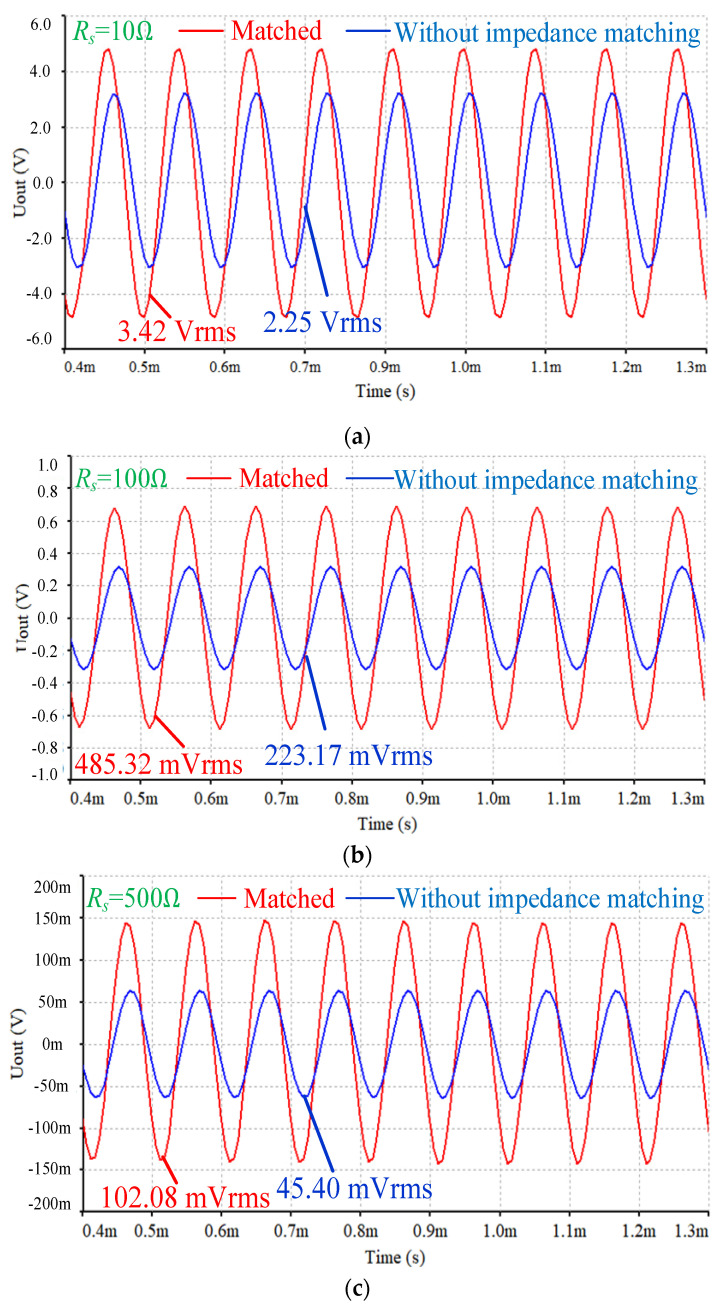

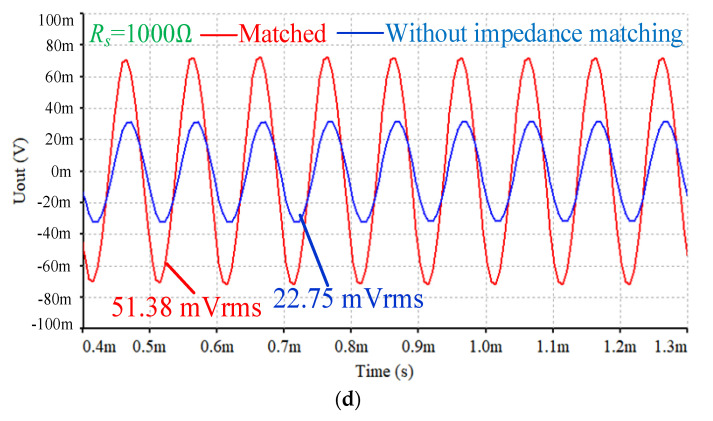
Simulation output waveform of sensor circuit model. (**a**) *R_s_* = 10 Ω; (**b**) *R_s_* = 100 Ω; (**c**) *R_s_* = 500 Ω; (**d**) *R_s_* = 1000 Ω.

Figure 7 illustrates the variation in the sensor’s output response with respect to changes in the equivalent resistance *R_s_*. The simulation results reveal a strong linear relationship between the sensor’s output and the reciprocal of the equivalent resistance, which is in line with the theoretical analysis presented in Equation (9). This linearity confirms that the sensor’s output is highly sensitive to changes in conductivity, as expected from the sensor’s design. Figure 8 shows the waveform of the sensor’s output at different *R_s_* values. As the equivalent resistance *R_s_* increases, the peak value of the output voltage gradually decreases, which aligns with the fundamental operating principle of the inductive conductivity sensor. Since the conductivity of the liquid (which is inversely related to *R_s_*) directly affects the strength of the induced current, a lower conductivity (i.e., higher *R_s_*) leads to a weaker induced current. This reduction in current causes a corresponding decrease in the output voltage amplitude. These findings demonstrate that the sensor’s output voltage accurately reflects the conductivity of the liquid, validating its potential for precise conductivity measurement in practical applications.

The impedance matching equivalent circuit model of the sensor was simulated using Multisim circuit simulation software. By utilizing the AC analysis function in the software, the equivalent impedance of the sensor at 10 kHz was found to be *R_m_* = 1.85 kΩ, *L_m_* = 10.14 mH. With a center frequency of 10 kHz, the sensor’s output response was compared between the cases with and without impedance matching. The bandwidth for the double tuning impedance matching was designed to range from 8 kHz to 12 kHz, and the input excitation voltage was maintained at *U*_1_ = 3 Vrms. Based on the previously mentioned formulas, the series matching capacitance *C_s_* and parallel matching capacitance *C_p_* were calculated. The simulation output waveforms of the sensor at different equivalent resistances *R_s_* after impedance matching are shown in Figure 8. According to the sensor’s equivalent circuit model, whether single tuning or double tuning impedance matching is used, the effect on the sensor’s output *U_out_* remains essentially the same. Therefore, in Figure 8, no distinction is made between the single tuning and double tuning impedance matching methods. A comparison with the case without impedance matching reveals a significant increase in the output waveform amplitude when impedance matching is applied. Under the same input conditions, the sensor with impedance matching demonstrates a notably stronger output signal. This substantial improvement in output amplitude confirms that impedance matching plays a crucial role in enhancing the sensor’s output performance, thereby validating its effectiveness in optimizing sensor operation.

**Figure 9:**
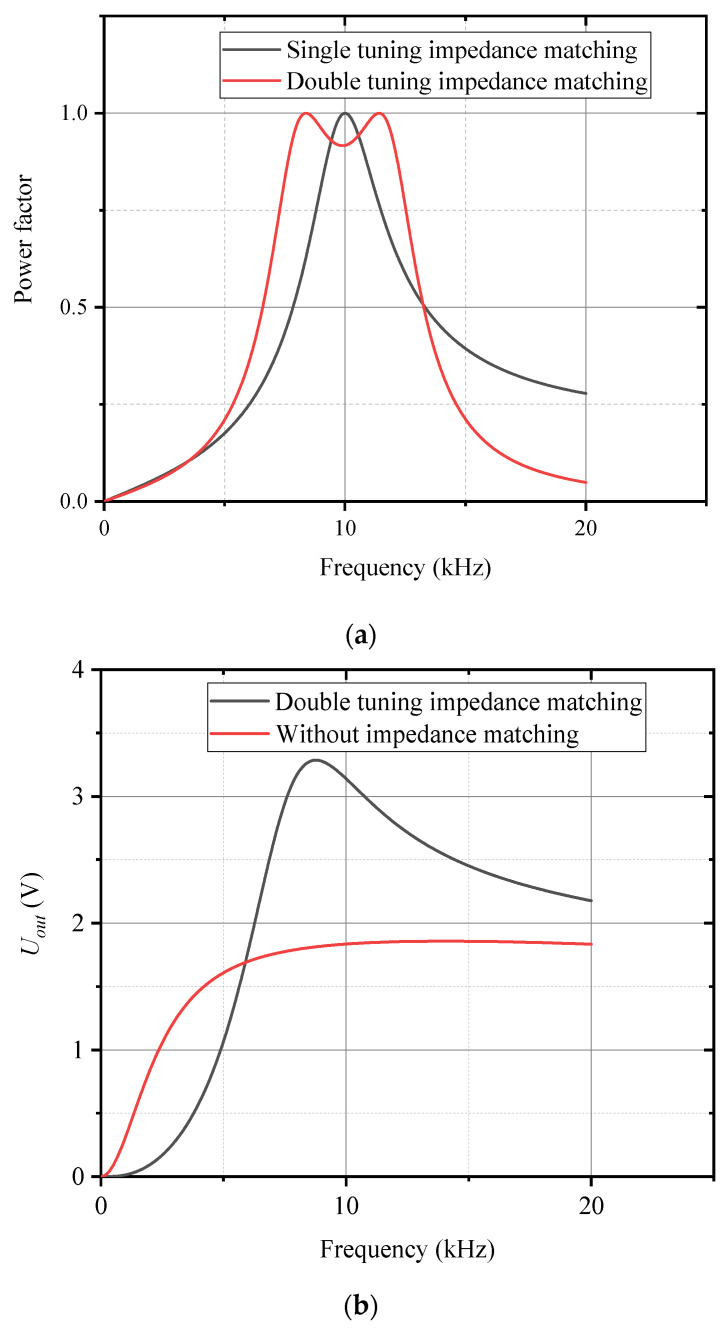
Frequency response of sensors. (**a**) Relationship between power factor and frequency; (**b**) relationship between output *U_out_* and frequency.

Compared to single tuning impedance matching, double tuning impedance matching achieves a higher power factor over a wider frequency range, effectively expanding the matching bandwidth. Figure 9a illustrates the load power factor as a function of frequency under different matching methods. From this figure, it is evident that single tuning impedance matching provides a power factor of 1 at the matching frequency point; however, the power factor rapidly decreases as the frequency deviates from the matching point. In contrast, by carefully designing the bandwidth for double tuning impedance matching, a flatter passband is obtained. This characteristic of wideband matching ensures that double tuning impedance matching maintains stable sensor operation across a broader frequency spectrum, minimizing performance degradation due to frequency shifts. Consequently, double tuning impedance matching is more advantageous for applications that demand high sensitivity and stable performance across varying frequencies. Figure 9b shows the frequency response curves of the conductivity sensor with and without impedance matching. It is evident that without impedance matching, the sensor’s output signal varies relatively smoothly with frequency with minimal amplitude variation. However, after introducing double tuning impedance matching, the output response of the sensor exhibits a pronounced dependence on frequency with the signal undergoing significant changes as the frequency increases. Further analysis based on the equivalent circuit model of the inductive conductivity sensor and the double tuning impedance matching network reveals that the output response undergoes not only strong frequency dependence but also notable nonlinearity, as shown in Figure 7. This indicates that while double tuning impedance matching enhances the sensor’s output signal, the relationship between the output signal and frequency becomes more complex within a specific frequency range, resulting in nonlinear changes in the output. Therefore, in practical applications, this nonlinear behavior must be taken into account, and additional calibration or compensation may be necessary to ensure the accuracy of measurements.

### 3.3. Performance Evaluation of Sensor Output

The sensitivity and linearity of the inductive conductivity sensor are critical evaluation metrics for assessing its output performance after incorporating an impedance matching network for wideband compensation. These parameters provide insights into the sensor’s ability to detect small variations in conductivity and the degree to which the sensor’s output maintains a proportional relationship with the measured conductivity. The following methods are used to calculate the sensor’s sensitivity and linearity:

(11) $$\left\{\begin{array}{c} Sens=\frac{U_{out}(G_{s}^{max}-U_{out}(G_{s}^{min})}{G_{s}^{max}-G_{s}^{min}}\\ Line=\frac{\Delta Y_{max}}{Y} \end{array}\right.$$

where *Sens* is the sensitivity of the sensor, *Line* is the linearity. $G_{s}^{min}$ is the lower limit of the conductivity measurement range, and $G_{s}^{max}$ is the upper limit of the conductivity measurement range. $U_{out}(G_{s})$ is the sensor’s output response function, and $U_{out}(G_{s}^{max})$ and $U_{out}(G_{s}^{min})$ are the sensor’s output responses at the upper and lower limits of the measurement range, respectively. $\Delta Y_{max}$ is the maximum deviation between the sensor calibration curve and the fitted straight line, and *Y* is the full-scale output of the sensor. Both *Sens* and *Line* are functions of the working frequency of the inductive conductivity sensor.

To comprehensively evaluate the overall performance of the sensor, both linearity and sensitivity are weighted according to their relative importance. The performance evaluation function that optimizes the overall sensor performance can be expressed as a weighted difference of the sensitivity and linearity:

(12) $$F_{total}=\alpha_{1}{Sens}^{*}-\alpha_{2}{Line}^{*}$$

where $F_{total}$ is the overall performance evaluation function. $\alpha_{1}$ is the weight assigned to the sensitivity and $\alpha_{2}$ is the weight assigned to the linearity. The weights $\alpha_{1}$ and $\alpha_{2}$ are chosen based on the relative importance of each factor in the specific application. For instance, if higher sensitivity is more crucial for the intended application, a larger weight can be assigned to the sensitivity term. Similarly, if maintaining linearity over the full measurement range is more important, the linearity weight can be increased. The values of $\alpha_{1}$ and $\alpha_{2}$ should satisfy the condition $\alpha_{1}+\alpha_{2}\;$= 1. The linearity and sensitivity of the sensor are normalized to bring them to a common scale, typically in the range of 0 to 1, ensuring that both metrics contribute proportionally to the overall evaluation:

(13) $$\left\{\begin{array}{c} {Sens}^{*}=\frac{Sens-{Sens}_{min}}{{Sens}_{max}-{Sens}_{min}}\\ {Line}^{*}=\frac{Line-{Line}_{min}}{{Line}_{max}-{Line}_{min}} \end{array}\right.$$

where ${Sens}^{*}$ and ${Line}^{*}$ are the normalized values of the sensitivity and linearity, respectively. ${Sens}_{min}$ and ${Sens}_{max}$ are the minimum and maximum values of the sensitivity across the measurement range. ${Line}_{min}$ and ${Line}_{max}$ are the minimum and maximum values of the linearity across the measurement range.

This ensures that both sensitivity and linearity are proportionally accounted for in the evaluation. By optimizing this performance evaluation function, the overall sensor performance can be maximized, simultaneously enhancing both sensitivity and linearity for improved measurement accuracy.

## 4. Experimentation Verification

### 4.1. Experimental Platform Construction

**Figure 10:**
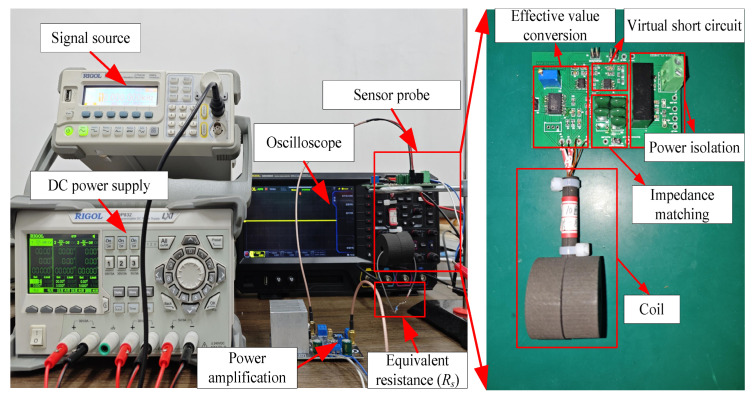
Experimental platform.

The experimental setup used in this work is shown in Figure 10. The main components of the platform include a signal generator, DC power supply, power amplification module, inductive conductivity sensor probe, oscilloscope, and other associated equipment. The inductive conductivity sensor probe consists of an excitation coil, sensing coil, impedance matching module, and effective value conversion module with its physical parameters corresponding to those listed in Table 1. The signal generator produces a sine wave excitation signal, which is amplified by the power amplification module to drive the excitation coil, generating an alternating magnetic field. For measurement purposes, a shorted resistor *R_s_* is used to simulate the conductivity of the liquid being measured. The sensing coil of the probe outputs the short-circuit current, which is then converted to the output voltage (*U_out_*) after passing through the virtual short-circuit measurement circuit, which is constructed using an operational amplifier. The output voltage, *U_out_*, is captured and displayed by the oscilloscope, and is further processed by the effective value conversion module for recording and analysis by the computer.

The impedance matching design aims to achieve a wide frequency band ranging from 8 kHz to 12 kHz. Using the sensor probe parameters listed in Table 1 and the impedance matching circuit design method outlined earlier, the matching capacitance values can be calculated. Taking measurement errors into account, the matching capacitance values are selected as *C_s_* = 36.5 nF, *C_p_* = 45.5 nF. The power amplification module is then adjusted to ensure that the effective value of the excitation voltage, *U*_1_, remains at 3 Vrms.

### 4.2. Experimental Results

**Figure 11:**
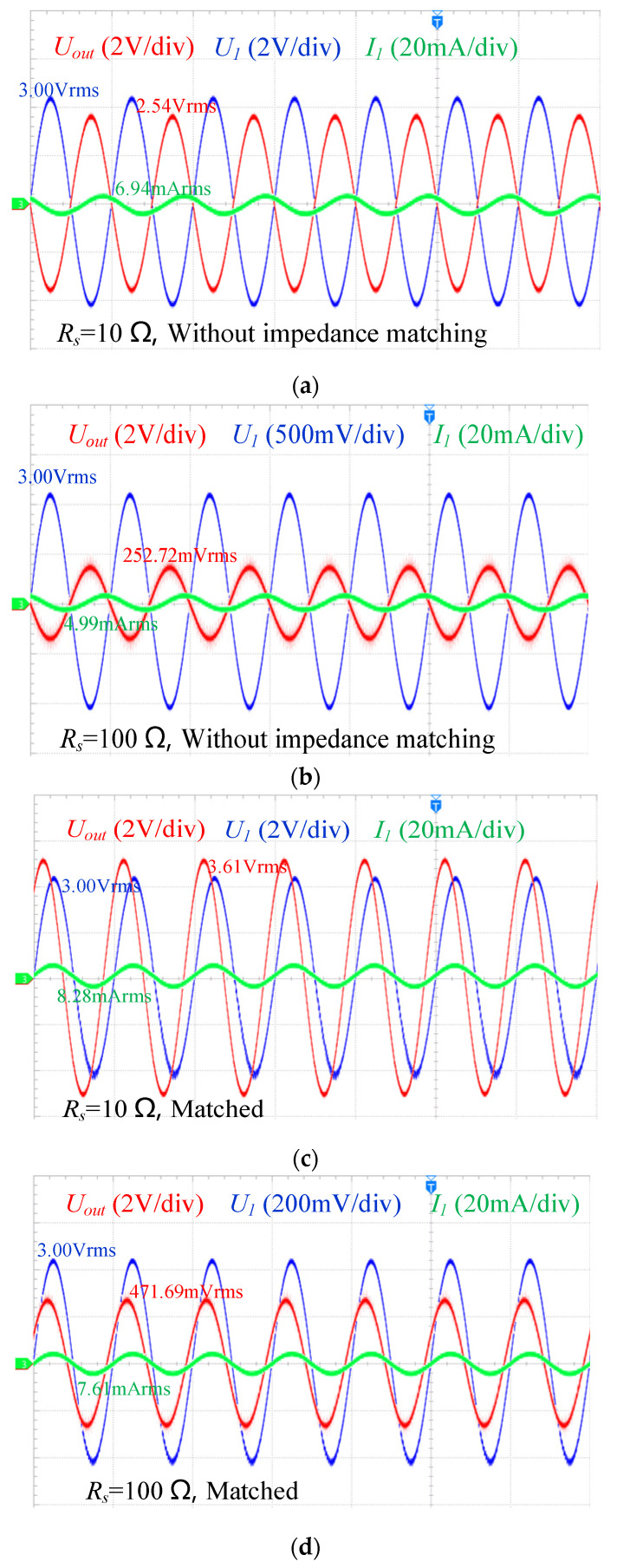
Sensor input current, input voltage, output voltage waveform. (**a**) *R_s_* = 10 Ω without impedance matching; (**b**) *R_s_* = 10 Ω without impedance matching; (**c**) *R_s_* = 100 Ω, matched; (**d**) *R_s_* = 100 Ω, matched.

First, the waveforms of the input current *I*_1_, input voltage *U*_1_, and output voltage *U_out_* of the sensor were measured under both impedance matching and without impedance matching conditions, as shown in Figure 11. A comparative analysis reveals that for the same conductivity measurement, the effective value of the output voltage *U_out_* is relatively low under without impedance matching conditions. However, after impedance matching, the effective value of *U_out_* increases significantly, particularly in low conductivity measurements, where the output signal grows by approximately 47%, thereby enhancing the sensor’s sensitivity. The primary purpose of impedance matching is to optimize the power transfer efficiency between the sensor and the external circuit. In the absence of impedance matching, there is a significant phase difference between the input current *I*_1_ and the input voltage *U*_1_, resulting in a low power factor (*cosφ*). After introducing impedance matching, the phase difference between the input current and input voltage approaches zero, causing the power factor to approach 1. This indicates a substantial improvement in power transfer efficiency. The Fast Fourier Transform (FFT) was applied to analyze the output waveform in the frequency domain. Using *R_s_* = 100 Ω as an example, the sensor’s output signal exhibited a signal-to-noise ratio (SNR) of 20.854 dB without impedance matching. After implementing impedance matching, the SNR increased to 23.1765 dB, representing an enhancement of 2.3711 dB. Overall, impedance matching enhances the power transfer efficiency, optimizes the power factor, and significantly improves the sensor’s output performance, enabling it to more effectively reflect changes in conductivity and enhancing its measurement capability in practical applications.

**Figure 12:**
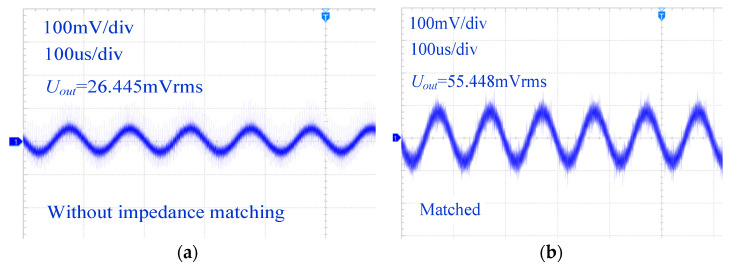
The sensor’s output waveform during measurements in the actual electrolyte solution. (**a**) Without impedance matching; (**b**) matched.

In this supplementary experiment, the inductive conductivity sensor was immersed in an actual electrolyte solution, differing from the previous experiment where an ideal *R_s_* was used to simulate the water loop. To address the limitations of using a resistor with only electronic conductivity, a solution with a conductivity of 784.1 µS/cm was prepared using table salt for practical validation. The experimental results revealed that due to the participation of complex ions in the electrolyte solution in forming the induced current, the sensor’s output voltage exhibited slightly lower waveform quality compared to the results obtained using *R_s_*, as it contained additional high-frequency harmonic components. However, the sinusoidal fundamental component of the sensor’s output remained well preserved. As shown in Figure 12, after implementing impedance matching, the sensor’s output improved significantly from 26.445 mVrms to 55.448 mVrms, demonstrating the effectiveness of the proposed impedance matching technique in enhancing the fundamental component and overall signal strength.

**Figure 13:**
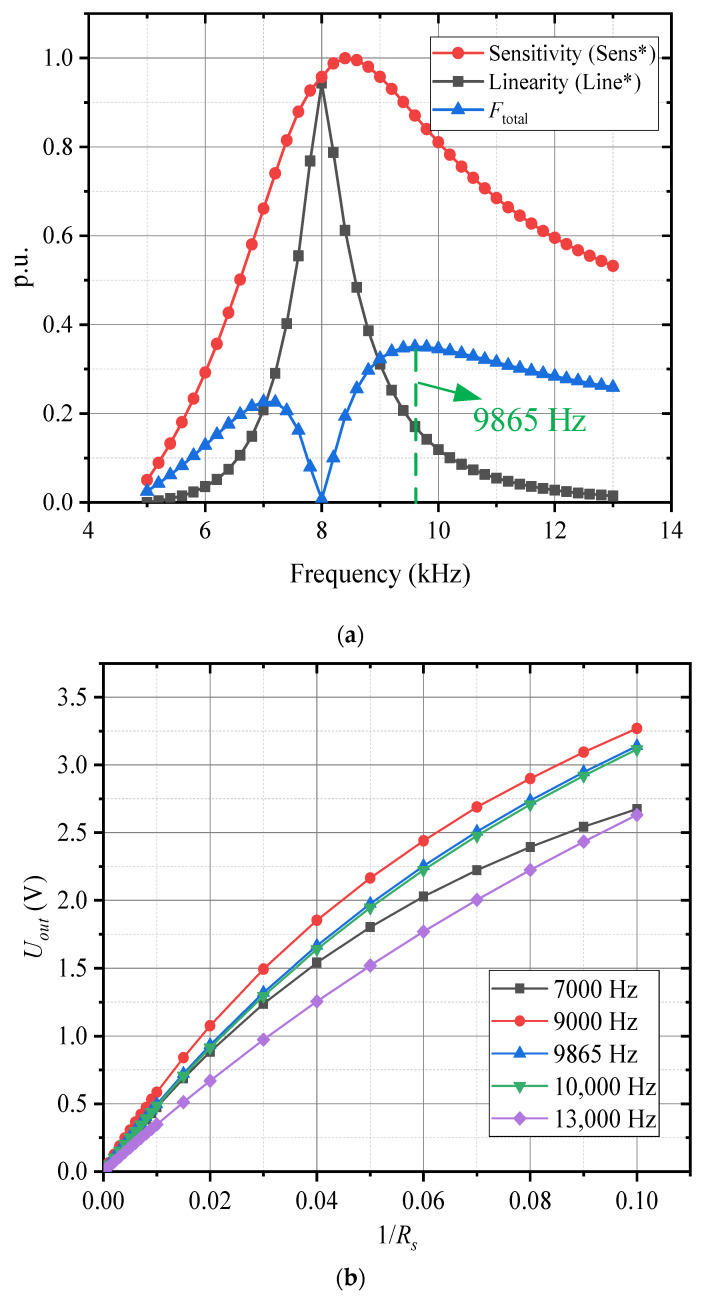
Sensor output response. (**a**) Linearity and sensitivity with frequency variation; (**b**) sensor output response curves at different frequencies after impedance matching.

After double tuning impedance matching, the sensor’s sensitivity and nonlinear error are shown in Figure 13a. As observed, both the sensitivity and linearity (nonlinear error) of the sensor increase with frequency, reaching their peak values at approximately 8 kHz. Beyond this point, the sensitivity gradually decreases, while the linearity quickly deteriorates. This suggests that within the matching frequency range, impedance matching effectively enhances the sensor’s sensitivity, making it more responsive to changes in conductivity. However, this improvement in sensitivity comes at the expense of increased nonlinear error, which compromises the sensor’s linearity.

To comprehensively evaluate the sensor’s linearity and sensitivity, the overall performance evaluation function was employed to balance these two factors. Figure 13a illustrates the variation in the performance evaluation function with frequency. As seen, the evaluation function undergoes significant changes across the frequency range, reaching a local peak at approximately 7 kHz before rapidly decreasing. It then reaches a minimum near 8 kHz and subsequently increases again, peaking at 9865 Hz. This trend indicates that the sensor’s overall performance is optimized within the matching frequency range with the best performance occurring at 9865 Hz. Figure 9b presents the sensor’s output response curves at different frequencies following impedance matching. It is evident that at each frequency, the response curve at 9865 Hz outperforms those at other frequencies. Compared to the without impedance matching case, the sensor’s sensitivity increased by approximately 30%. The output curve at this frequency exhibits high sensitivity, allowing the sensor to respond more effectively to changes in conductivity while maintaining good linearity throughout the entire test range, ensuring accuracy and consistency in the measurement results.

## 5. Conclusions

In this paper, an inductive conductivity sensor with double tuning impedance matching has been designed and evaluated for enhanced sensitivity and linearity. The impedance matching network significantly improves the sensor’s output performance with a notable increase in sensitivity—particularly at low conductivity measurements. Through the use of both theoretical analysis and experimental verification, it was demonstrated that impedance matching not only increases the output voltage but also optimizes the power transfer efficiency, achieving a higher power factor and a broader frequency range for the sensor’s operation. The results show that double tuning impedance matching effectively broadens the sensor’s frequency response with the best performance observed at a frequency of 9865 Hz. However, while the sensitivity is improved, the matching process also introduces nonlinear errors, which affect the sensor’s linearity. To assess the trade-off between sensitivity and linearity, a performance evaluation function was developed, highlighting that the sensor’s optimal performance occurs within the matching frequency range. The sensor’s sensitivity increases by approximately 30% compared to the without impedance matching case, demonstrating its enhanced capability to detect conductivity changes with greater precision.

## Figures and Tables

**Table 1:** Structural parameters of sensor probe.

Turns (*N*_1_ = *N*_2_ = *N*)	Core Dimension (*r*_1_ × *r*_2_ × *t*)	Magnetic Core Permeability (*μ_r_*)
10	16 mm × 25 mm × 10 mm	80,000

## Data Availability

The original contributions presented in this study are included in the article. Further inquiries can be directed to the corresponding author.
